# EGFR-mutated lung adenocarcinoma with choroidal oligometastasis during treatment with gefitinib: a case report

**DOI:** 10.1007/s13691-023-00653-3

**Published:** 2024-03-06

**Authors:** Takafumi Hashimoto, Atsushi Osoegawa, Miyuki Abe, Ryoko Oki, Takashi Karashima, Yohei Takumi, Kosuke Kamada, Michiyo Miyawaki, Kenji Sugio

**Affiliations:** 1 Department of Thoracic and Breast Surgery, Faculty of Medicine, Oita University, 1-1 Idaigaoka, Hasama-Machi, Yufu, Oita 879-5593 Japan; 2 Department of Ophthalmology, Faculty of Medicine, Oita University, 1-1 Idaigaoka, Hasama-Machi, Yufu, Oita 879-5593 Japan

**Keywords:** EGFR-positive non-small cell lung cancer, Choroidal metastasis, E709K, Oligometastasis

## Abstract

The patient was a 74-year-old woman who was diagnosed with lung adenocarcinoma, clinical Stage IIIA. Induction chemoradiation was performed followed by right upper lobectomy and lymph node dissection. Because of positive pleural effusion cytology, which was proven after surgery, the patient was diagnosed with pathological Stage IVA with EGFR L858R mutation. At 17 months after the administration of gefitinib, left choroidal metastasis appeared. Stereotactic irradiation and ruthenium small-beam radiation were effective; however, the metastatic lesion showed regrowth 7 months after these treatments. Because the patient’s choroidal oligometastasis was resistant to conservative therapy, left ophthalmectomy was performed. EGFR mutations (L858R and E709K) were detected in the resected choroidal tumor. The patient continued to take gefitinib. However, a neoplastic lesion developed on the optic nerve adjacent to the resected posterior eye segment. The lesion was treated with stereotactic radiation, gefitinib was switched to afatinib 30 mg, and the patient remains alive and disease free for 11 months.

## Introduction

Lung cancer metastasis of the choroid is rarely found because it is difficult to diagnose on imaging exams, and many cases are asymptomatic. A review of autopsy cases that included cancers other than lung cancer confirmed choroidal metastases in 12% of cases, suggesting latent asymptomatic metastasis [1].

We treated a patient with choroidal metastasis of EGFR-mutant lung cancer during gefitinib treatment. We also analyzed genetic mutations using tumor specimens from the primary lung lesion, metastatic choroidal lesion and blood; then we will discuss the mechanisms of choroidal resistance to EGFR-TKI treatment and the mechanism of oligo-resistance in the choroid.

## Case report

**Fig. 1:**
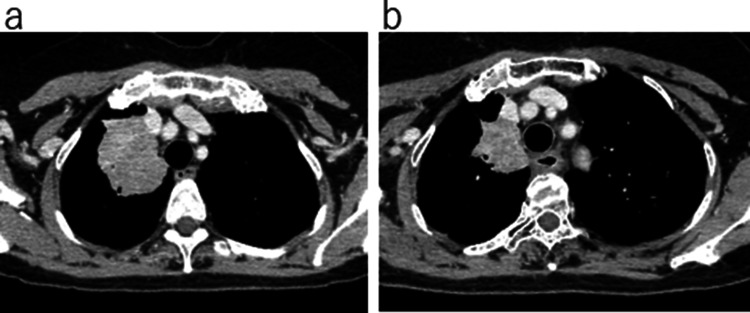
**a** CT, and PET-CT images before induction chemoradiation therapy. Chest CT scan showed a 56 mm right upper lobe mass shadow; PET-CT showed FDG accumulation in a lung mass and #4R lymph node. **b** Images after induction chemoradiation therapy

A 74-year-old female never-smoker visited our hospital after a lung mass was detected in the right upper lung field by chest X-ray. Chest CT revealed an irregular mass of 56 mm in diameter in the right upper lobe, which was suspected to invade the right brachiocephalic vein. The #4R and #10 lymph nodes were enlarged and fluorodeoxyglucose positron emission tomography (FDG-PET) showed positive uptake in the lung mass and the #4R and #10 lymph nodes; however, no distant metastasis was observed (Fig. 1A). A biochemical examination showed elevated CEA (47.7 ng/ml). EBUS-TBNA of #4R was performed; then adenocarcinoma was detected. Because the clinical stage was determined to be cT4N2M0 (Stage IIIB), induction chemoradiotherapy with 2 cycles of cisplatin/S-1 combination therapy and 40 Gy radiation, concomitantly for the primary lesion and mediastinal lymph node, was performed. The treatment response was classified as a PR, with a 43% reduction in size; however, invasion of the right brachiocephalic vein was still suspected based on CT; thus, the preoperative clinical stage was considered to be ycT4N2M0 (Stage IIIB) (Fig. 1B). Intraoperative findings showed no invasion to the right brachiocephalic vein; then right upper lobectomy and lymph node dissection were performed. Although there was no pleural dissemination macroscopically, cancer cells were detected in the pleural effusion, which was proven after surgery (R1). The pathological diagnosis was adenocarcinoma (acinar predominant) with metastasis to the #4R and #12u lymph nodes. The pathological stage was determined to be ypT2bN2M1a (Stage IVA). Genetic testing using both Cobas and Oncomine Dx Target Tests (Thermo Fisher Science) showed L858R EGFR mutation. The immunohistochemical expression of PD-L1 (22C3, DAKO) in the surgical specimen was 20%. The CEA level normalized after surgery. Gefitinib (250 mg) was administered due to stage IVA disease with positive pleural fluid cytology. The reason gefitinib was chosen as the first-line treatment in this case was because it was before the approval of osimertinib.

**Fig. 2:**
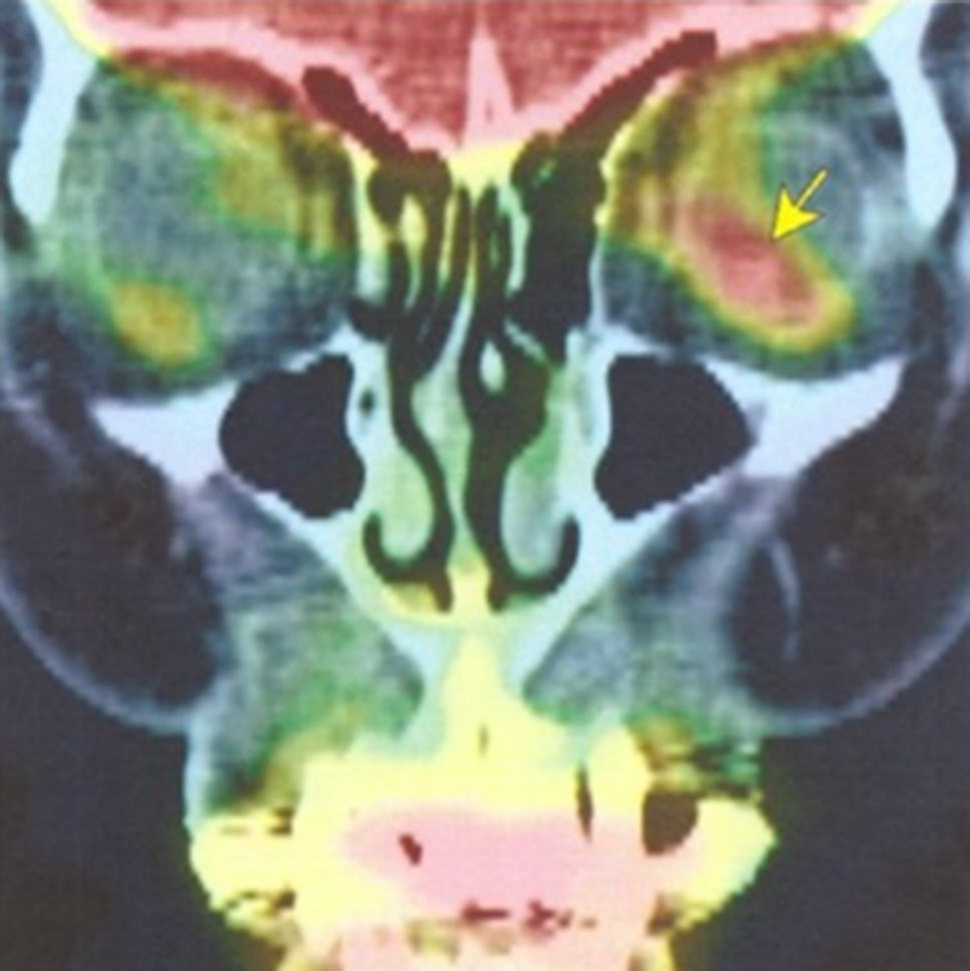
PET-CT at the time of recurrence of choroidal metastasis showed FDG accumulation in the posterior eye

**Fig. 3:**
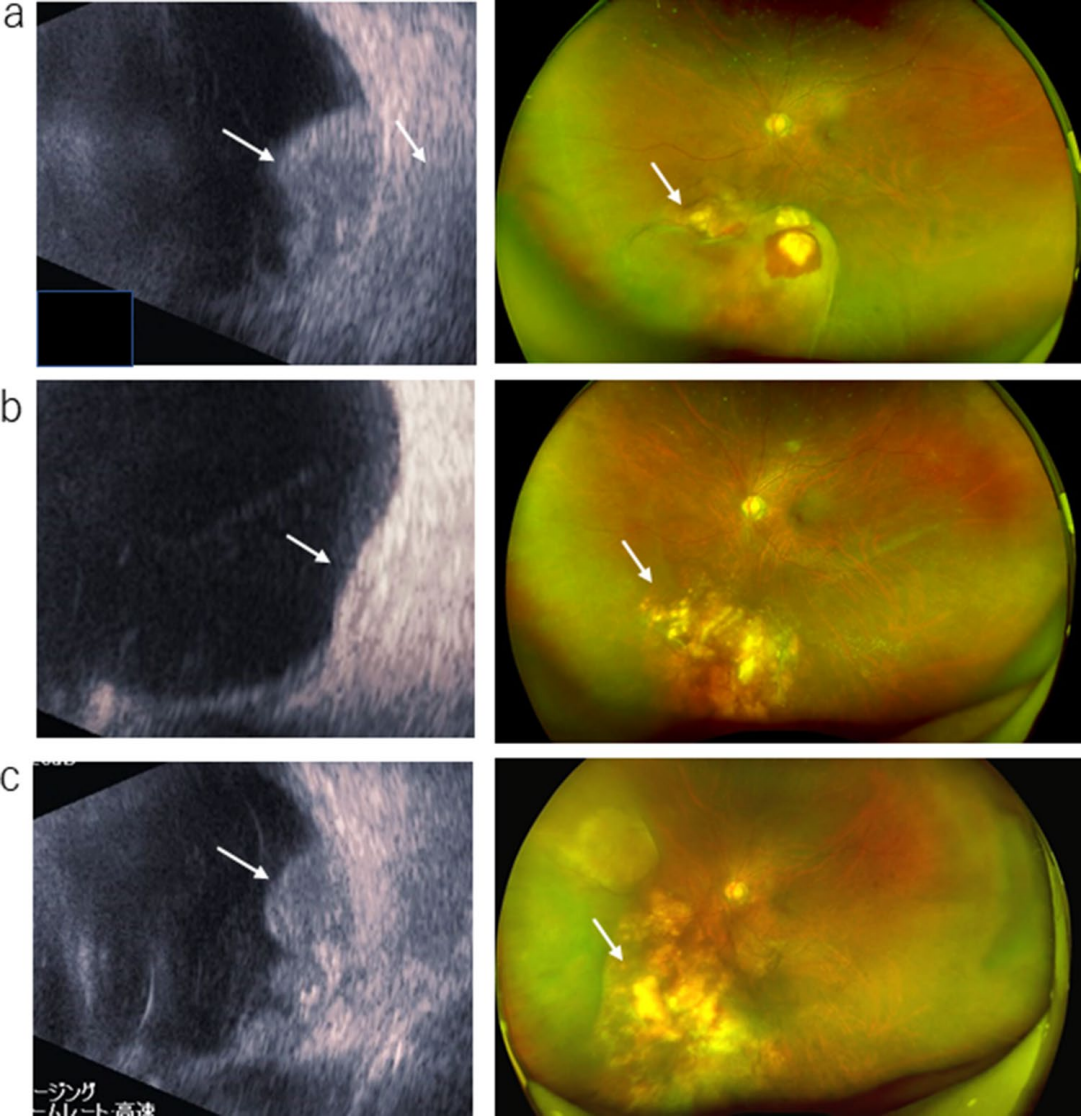
Fundus and echocardiographic findings **a** at initial ophthalmologic examination, **b** after radiotherapy, **c** before oophorectomy

At 17 months after starting gefitinib treatment, the patient became aware of pain around the left eye without visual field impairment. An ophthalmological examination showed left choroidal metastasis using echography and fundus findings. PET-CT showed the uptake of FDG in the posterior part of the left eye (Figs. 2 and 3A). A plasma EGFR test detected L858R, but no other mutations, including T790M. Stereotactic irradiation of 30 Gy/10 fr was performed, and tumor shrinkage and ocular pain were observed (Fig. 3B).

Gefitinib was continued because the only recurrent lesion observed on CT was that of the left choroid; however, the metastatic lesion of the choroid showed regrowth 5 months later. The patient received ruthenium small-beam radiation therapy (80 Gy), which again resulted in tumor shrinkage. At 5 months after small-beam radiation therapy, echography showed tumor regrowth and photodynamic therapy and intravitreal injection of aflibercept were administered; however, however, at 7 months after the intravitreal injection of aflibercept, the tumor continued to grow. A plasma EGFR test detected L858R, but not T790M, which was the same result as before.

**Fig. 4:**
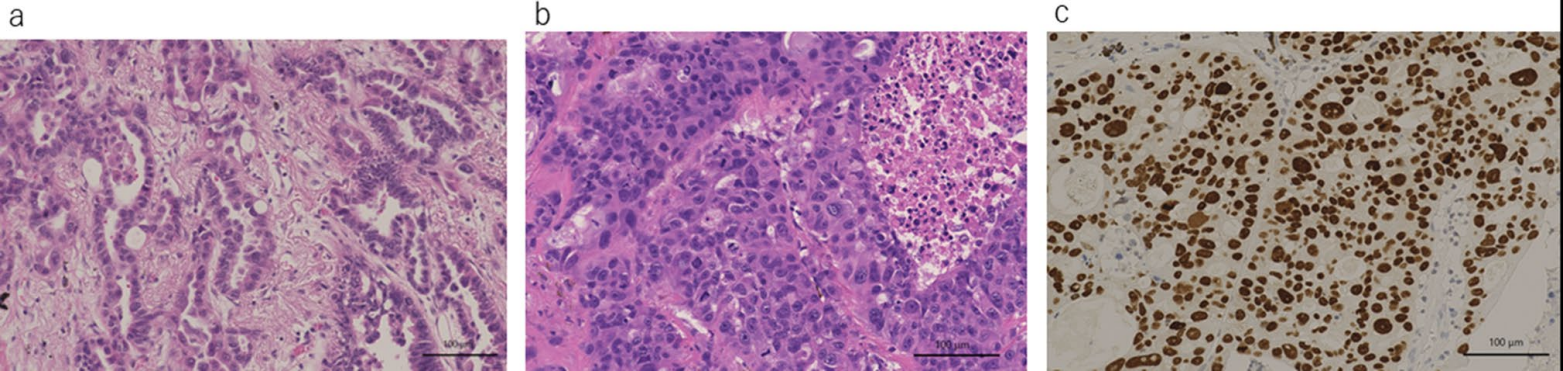
Pathologic finding of the primary lesion and choroid. **a** HE staining of primary lung lesion, **b** HE staining of choroid, **c** TTF-1 staining of choroid. Choroidal lesions showed atypical cells that were positive for TTF-1 staining

Because the choroid was the organ of oligo metastasis and was resistant to repeated conservative therapy, the patient underwent left ophthalmectomy (3 years and 4 months after the initiation of gefitinib) (Fig. 3C). The pathological diagnosis of the choroid tumor was adenocarcinoma, which was positive for TTF-1 and Napsin A (Fig. 4). An Oncomine Dx Target test (Thermo Fisher Science) of the resected eye specimen detected EGFR mutations, specifically L858R and E709K (which was newly detected), but was negative for T790M. Because CT revealed no recurrent lesions other than the choroid lesion, the patient continued to take gefitinib. However, a neoplastic lesion developed on the optic nerve adjacent to the resected posterior eye segment at 21 months after ophthalmectomy. Stereotactic radiation targeting the lesion was administered, gefitinib was switched to afatinib 30 mg, and the patient remains alive and disease free 11 months later.

## Discussion

The choroid is a rare target of metastasis; however, it is detected in examinations focusing on choroidal metastases, including asymptomatic metastases. The rate of choroidal metastasis from several cancers is reported to be 4–10% in autopsy tissues [1–4]. Kreusel et al. reported that screening of the eyes by funduscopy and ultrasonography in 84 consecutive metastatic lung cancer patients managed between 1995 and 1998 revealed 6 patients (7%) with choroidal metastasis, all of whom were asymptomatic [5]. Bouchez et al. reported screening of the eyes, using MRI, of 83 consecutive metastatic EGFR-mutant NSCLC patients between 2015 and 2018 and detected 7 patients (8%) with choroid metastasis, six of whom had visual disturbance [6]. Although these two reports were from a different era, the examination methods, genetic mutations of lung cancer, and results of choroidal metastasis rates were similar. Choroidal metastasis is more likely to be detected in lung cancer patients than is experienced in the clinical setting when aggressive investigation is performed. It is also interesting that although the present case had EGFR-mutant lung cancer, symptomatic choroidal metastasis was more common in EGFR-mutant lung cancer. However, these two reports investigated lung cancer with distant metastasis, and cases of choroidal oligometastasis, such as the present case, are rare.

Most metastases to the eye occur at the choroid (88%), followed by the iris (10%) and ciliary body (2%). It was bilateral in 18% of cases. Breast cancer is reported to be the most common primary cancer (40–53%), followed by lung cancer (20–29%) [7].

The Oncomine Dx Target Test, an NGS-based genetic test, was performed on surgically resected primary lung specimens, which revealed no E709K mutation prior to gefitinib treatment. E709K is reported to account for 0.5% of EGFR-positive lung cancers in the COSMIC database and 0.3% in the report by Kobayashi et al. [8]. As for E709K, EGFR-TKIs are expected to have some efficacy, although it has been excluded from the major global phase III trials of EGFR-TKI. Kobayashi et al. also reported that second- or third-generation TKIs are more effective for lung cancer with E709K in comparison to first-generation TKIs, with IC50s as high as 187 nM for gefitinib and 215 nM for erlotinib versus 0.7 nM for afatinib and 62 nM for osimertinib [8].

E709K, located in exon 18, is known as a mutation with low sensitivity to EGFR-TKIs. There are no reported cases demonstrating the effectiveness of second- or third-generation EGFR-TKIs for this mutation [8–10]. However, in LUX-LUNG trial, the pivotal study for afatinib, one case of complete response (CR) was reported in a patient with the compound mutation L858R + E709K (the same mutation pattern as in this case) [11]. Additionally, in the retrospective UNICORN trial, which assessed the effectiveness of osimertinib against uncommon mutations, PFS (progression-free survival) of 5.5 and 8.8 months was reported in two cases with the G719A + E709K compound mutation [12]. These findings contribute to the understanding of the potential efficacy of second- or third-generation EGFR-TKIs against the E709K mutation.

The treatment of choroidal metastases includes radiation therapy, photocoagulation, photodynamic therapy (PDT), and ophthalmectomy, but it is important to consider the patient’s prognosis and QOL. Drug transport to the posterior segment of the eye is limited by mechanisms, such as the blood–retinal barrier, and the effects of systemic chemotherapy are believed to be poor, although better responses to molecularly targeted agents (e.g., afatinib and osimertinib) have been reported [13, 14].

Oligometastasis is a state in which cancer patients have a limited number of metastases. Local treatment (e.g., surgery or radiation therapy) has been reported to prolong the prognosis, even in patients with stage IV distant metastases [15, 16]. In the present case, local treatment with radiotherapy was performed first. In this case, we selected ophthalmectomy for a choroidal lesion that was refractory to radiotherapy and small-beam radiation therapy because it was oligometastasis. The second-generation EGFR-TKI, afatinib, may be effective for patients with E709K mutation.

## Conclusion

Choroidal metastasis appeared in a patient with lung cancer with an EGFR L858R mutation during treatment with the first-generation EGFR-TKI, gefitinib. Ophthalmectomy was performed after the failure of radiotherapy. EGFR E709K mutation was detected in the choroidal metastatic lesion, which was not detected in the primary tumor. The second-generation EGFR-TKI, afatinib, may be effective for patients with EGFR E709K mutation.

## Data Availability

The dataset generated and analyzed during the current study are available from the corresponding author on reasonable request.

## References

[1] Bloch RS, Gartner S (1971). The incidence of ocular metastatic carcinoma. Arch Ophthalmol.

[2] Ferry AP, Font RL (1974). Carcinoma metastatic to the eye and orbit. I. A clinicopathologic study of 227 cases. Arch Ophthalmol.

[3] Albert DM, Rubenstein RA, Scheie HG (1967). Tumor metastasis to the eye. I. Incidence in 213 adult patients with generalized malignancy. Am J Ophthalmol.

[4] Shields CL, Shields JA, De Potter P (1997). Plaque radiotherapy for the management of uveal metastasis. Arch Ophthalmol.

[5] Kreusel KM, Stange TWM, Bornfeld N (2002). Choroidal metastasis in disseminated lung cancer: frequency and risk factors. Am J Ophthalmol.

[6] Bouchez C, Pluvy J, Soussi G (2020). Epidermal growth factor receptor-mutant non-small cell lung Cancer and Choroidal metastases: long-term outcome and response to epidermal growth factor receptor tyrosine kinase inhibitors. BMC Cancer.

[7] Shah SU, Mashayekhi A, Shields CL (2014). Uveal metastasis from lung cancer: clinical features, treatment, and outcome in 194 patients. Ophthalmology.

[8] Kobayashi Y, Mitsudomi T (2016). Not all epidermal growth factor receptor mutations in lung cancer are created equal: perspectives for individualized treatment strategy. Cancer Sci.

[9] Kohsaka S, Nagano M, Ueno T (2017). A method of high-throughput functional evaluation of EGFR gene variants of unknown significance in cancer. Sci Transl Med.

[10] Ciardiello F, Tortora G (2008). EGFR antagonists in cancer treatment. N Engl J Med.

[11] Yang JC, Sequist LV, Geater SL (2015). Clinical activity of afatinib in patients with advanced non-small-cell lung cancer harbouring uncommon EGFR mutations: a combined post-hoc analysis of LUX-Lung 2, LUX-Lung 3, and LUX-Lung 6. Lancet Oncol.

[12] Bar J, Peled N, Schokrpur S (2023). Uncommon EGFR mutations: international case series on efficacy of osimertinib in real-life practice in first-line setting (UNICORN). J Thorac Oncol.

[13] Zhou HP, Tanaka R, Tsuji H (2020). Therapeutic changes in bilateral choroidal metastasis from non-small cell lung cancer with response to afatinib: a case report. Ocul Immunol Inflamm.

[14] Dall'Olio F, Ruatta C, Melotti B (2017). Response to osimertinib in choroidal metastases from EGFRmt T790M-positive non-small cell lung adenocarcinoma. J Thorac Oncol.

[15] Gao XL, Zhang KW, Tang MB (2017). Pooled analysis for surgical treatment for isolated adrenal metastasis and non-small cell lung cancer. Interact Cardiovasc Thorac Surg.

[16] Gomez DR, Tang C, Zhang J (2019). Local consolidative therapy vs. maintenance therapy or observation for patients with oligometastatic non-small-cell lung cancer: longterm results of a multi-institutional, phase II, randomized study. J Clin Oncol.

